# Towards equity-focused intersectoral practice (EquIP) in children’s environmental health and housing: the transformational story of RentSafe

**DOI:** 10.17269/s41997-018-0094-x

**Published:** 2018-06-15

**Authors:** Erica Phipps, Jeffrey R. Masuda

**Affiliations:** 1 Canadian Partnership for Children’s Health and Environment (CPCHE), Toronto, ON Canada; 2 School of Kinesiology and Health Studies, Queen’s University, 28 Division Street, Kingston, ON K7L 3N6 Canada; 3 Centre for Environmental Health Equity (CEHE), Kingston, Canada; 4 Department of Geography, Queen’s University, 28 Division Street, Kingston, ON K7L 3N6 Canada

**Keywords:** Health equity, Intersectoral practice, Environmental health, Children, Housing, Knowledge translation, Health promotion, Social justice, Équité en santé, Pratique intersectorielle, Santé environnementale, Enfants, Logement, Application des connaissances, Promotion de la santé, Justice sociale

## Abstract

**Setting:**

This paper chronicles the transformational process through which a national intersectoral collaboration, the Canadian Partnership for Children’s Health and Environment (CPCHE), came to embrace a more upstream, equity-based focus in its mandate to advance children’s environmental health.

**Intervention:**

After 15 years of working within a conventional, evidence-informed approach to health promotion and policy advocacy, in 2010–2013, CPCHE had the opportunity to collaborate on the development of equity-focused knowledge translation (EqKT). EqKT is a relational approach to knowledge practices that challenges intersectoral actors to work to uncover biases and limitations within their own institutional paradigms and professional practices that constrain their capacity to address population health inequities.

**Outcome:**

The ensuing transformation towards equity-focused intersectoral practice led CPCHE to create an intersectoral initiative called RentSafe. Conceptually and operationally, RentSafe provides an intersectoral space within which the grounded expertise of people with experience of unhealthy and undignified housing provides a roadmap for public health and other practitioners to critically explore professional and institutional blind spots and barriers. With RentSafe as its watershed moment, CPCHE is shifting from a top-down “for whom” orientation to an authentically engaged “with whom” approach that seeks to work integrally with community partners to expose and challenge systemic roots of health inequity.

**Implications:**

The transformational story of CPCHE underscores the competencies needed for public health professionals to acknowledge the sources of our own biases and limitations as a necessary first step in equity-focused intersectoral practice (EquIP). It also affirms the value of working in partnership with those who experience the environmental health inequities that such efforts seek to address.

## Résumé

**Contexte:**

Cet article relate les détails du processus transformationnel par lequel une alliance nationale intersectorielle d’organisations, le Partenariat canadien pour la santé des enfants et l’environnement (PCSEE), en est venue à centrer davantage sa mission, qui consiste à promouvoir la santé environnementale des enfants, sur l’équité et une approche en amont.

**Intervention:**

Après 15 années de travail guidé par une approche traditionnelle fondée sur des données probantes en matière de promotion de la santé et de défense des politiques, le PCSEE a eu l’occasion de collaborer, entre 2010 et 2013, à la conception d’une approche d’application des connaissances axée sur l’équité nommée EqKT. EqKT se veut une approche relationnelle des pratiques d’application de connaissances qui invite les acteurs intersectoriels à mettre en lumière les limites et les préjugés inhérents à leurs paradigmes organisationnels et leurs pratiques professionnelles, et qui sont susceptibles de restreindre leur capacité à s’attaquer aux iniquités sur le plan de la santé au sein de la population.

**Retombée:**

La transition vers une pratique intersectorielle axée sur l’équité qui découle de ce processus transformationnel a conduit le PCSEE à créer une initiative intersectorielle appelée LogementSain. Sur les plans conceptuel et opérationnel, l’initiative LogementSain offre un espace intersectoriel où l’expertise acquise sur la base d’expériences de personnes ayant vécu des conditions de logement insalubres et indignes sert de guide aux intervenants en santé publique et aux autres praticiens afin qu’ils jettent un regard critique sur les angles morts et les barrières professionnelles et organisationnelles. Alors que l’initiative LogementSain arrive à un moment décisif, le PCSEE passe d’une approche descendante de type « pour qui » à une approche véritablement engagée de type « avec qui ». Cette dernière entend travailler en intégrant des partenaires communautaires afin d’exposer les racines systémiques des iniquités en matière de santé et de s’y attaquer.

**Implications:**

Le processus transformationnel vécu par le PCSEE met en évidence les compétences nécessaires aux professionnels du domaine de la santé publique afin qu’ils reconnaissent, en guise de préalable à la mise en place d’une pratique intersectorielle axée sur l’équité (ÉquIP), les origines de nos préjugés et de nos limites. Ce processus confirme également l’importance de travailler en partenariat avec les personnes victimes des iniquités sur le plan de la santé environnementale auxquelles de tels efforts tentent de remédier.

Environmental conditions during early life influence health and development and, ultimately, children’s lifelong prospects for well-being (Cooper et al. 2011; Landrigan et al. 2002; Wigle 2003). Unsafe living conditions, including toxic exposures, are among the environmental factors that can impair health, especially during the vulnerable stages of fetal and child development (Chen et al. 2014; Landrigan et al. 1999; Waterston et al. 2015; Weitzman et al. 2013). Children in low-income and other marginalized circumstances typically have disproportionately higher exposures and often have greater susceptibility to harm, setting them onto lifelong trajectories that exacerbate environmental health inequities (Cooper et al. 2011; Lanphear et al. 2002; Masuda et al. 2008).

Given the high stakes, organizations including those in the non-profit sector engage in a range of knowledge translation (KT) and advocacy efforts that target environmental exposures during the perinatal period and childhood. The Canadian Partnership for Children’s Health and Environment (CPCHE) is an exemplar of such effort. Established in 2001, CPCHE is a collaborative of organizations working in public health, medicine, environmental protection, child care, and disability advocacy that integrate their respective capacities to advance children’s environmental health protection in Canada.

Effecting change in support of children’s environmental health is challenging, often piecemeal and slow. As scientific knowledge of the health consequences of toxicants emerges, many respond with substance-specific and often consumer-oriented efforts—getting bisphenol A out of baby bottles, for example, or calling for better labeling of chemicals in personal care products. While such efforts succeed in galvanizing public attention and may lead to some reduction in exposures, they have largely overlooked the structural conditions—economic disparities, racism, patriarchal and colonial legacies, class-based stigma, and other marginalizing forces—that can prevent such benefits from reaching some communities.

This failure to take on environmental health inequities is not because of a lack of recognition that they exist (NCCDH 2013; PHAC 2008a) nor a lack of good intention among people on the frontlines of health promotion. Rather, it reflects a long pattern of retrenchment into a biomedical, evidence-based approach to health promotion that tends towards measures to effect individual behaviour change (e.g., personal actions to reduce harmful exposures) over efforts to address upstream drivers, such as reducing poverty and correcting environmental injustice (Masuda et al. 2010). This narrowing has eclipsed the more holistic framing that had been a hallmark of public health in Canada (Hancock 2011). Consequently, the terrain where environmental health, social justice, and advocacy intersect can feel unfamiliar, intimidating, or even threatening to those who are accustomed to working within prevailing evidence-based paradigms.

Our story is about how an intersectoral collaborative, CPCHE, set its course into this uncertain terrain, and what we have learned thus far.

## Intervention (or transformational trigger?)

The story begins 7 years ago with a collaboration between CPCHE and the Centre for Environmental Health Equity (CEHE), a consortium of researchers and practitioners, led by Jeff Masuda (JM), that was created to reposition academic research to better serve the needs and priorities of communities facing environmental health inequities. In 2010, CEHE began developing a training approach to help transform early career children’s environmental health practitioners into “Knowledge Leaders” for environmental health equity, and invited CPCHE to take part as co-organizer. The resulting Knowledge Leaders training offered a reflexive approach to co-learning intended to open up new ways of conceiving of and addressing children’s environmental health inequities. It was predicated on an emergent framework for knowledge practices called equity-focused knowledge translation (EqKT) that explicitly targets the conditions that prevent multiple ways of knowing from being given equitable status within professional intersectoral practices (Masuda et al. 2014).

This collaboration between Erica Phipps (EP) and JM—one the director of a national children’s environmental health collaborative and the other a community-engaged critical public health social scientist—began as a shared pursuit of ways to bring equity-focused practice into the realm of children’s environmental health promotion. It ended up triggering an ongoing transformation for EP and the start of a new chapter for CPCHE.

### What does it mean to be equity-focused?

To be equity-focused is to take up the challenge of questioning the ways in which we as professionals conceive of and approach “knowledge” in our work, as a critical first step. It requires us to recognize that knowledge is a form of power and to confront our tendency to allow institutionally generated science to eclipse other ways of knowing in our everyday work. Second, rather than accepting racism, classism, patriarchy, and colonialism as a static “context” to which our work must be adapted, a commitment to equity-focused practice compels us to surface, challenge and, ultimately, change unjust societal conditions.

To make this shift, we must reflect on the places we work, the ways we communicate, and the solutions that we propose. In doing so, we may start to see how inequities can be reinforced within institutions and funding streams, how the ways in which we communicate reflect the privileging or discounting of arenas in which knowledge is conveyed (e.g., professional conferences versus street protests), and how these power-knowledge dynamics influence with whom we work as partners to define problems and solutions.

### The Knowledge Leaders’ experiment

In August 2011, CEHE and CPCHE brought together 20 early career children’s environmental health practitioners from across Canada, including people working in social services, policy analysis, epidemiology, Indigenous rights, arts-based activism, and environmental health promotion, for an immersive experiment in equity-focused knowledge practices. During an intensive week spent together outside of our professional environments and in green spaces, homeless drop-ins, reserves, and schools, all of the Knowledge Leaders participants—including EP and JM—challenged ourselves to recognize our own biases, privileges, and blind spots. We worked to uncover the ways in which our conventional ways of thinking and working can act as barriers to partnering with those (e.g., in community settings) who may perceive, speak about, and approach environmental health issues in ways that differ from our own.

It is fair to say that the experiment worked. The ripple effects of the training are still in evidence in the form of experientially grounded collaborations led by Knowledge Leaders. What was not predicted was the impact that the experience would have on those of us who worked as co-conveners. The Knowledge Leaders’ experiment set into motion a further integration of CEHE and CPCHE’s work, which has fostered a more practice-based orientation to JM’s research endeavours in environmental health equity. For EP, it triggered a significant rethinking of CPCHE’s intersectoral work in the 7 years since.

## Outcomes

Equipped with new ways of understanding environmental health inequities and motivated by critical self-reflection, EP began to apply an equity-focused approach in leading CPCHE’s work. With the guidance and support of JM and several CPCHE partners (one of whom is a Knowledge Leaders’ alumna), EP set about reorienting CPCHE’s work towards a more authentically engaged, upstream approach. This journey brought CPCHE into the realm of housing, poverty, and social injustice and led to the launch of a rapidly expanding intersectoral initiative called RentSafe.

CPCHE had long prioritized the role of home environments in children’s health. Our popular “Top 5 Tips” campaign for creating healthy home environments for kids, our “Healthy Retrofits” initiative, and our years of advocacy and outreach on indoor environmental hazards, such as lead, flame retardants, plasticizers, and radon, all reflect our concern about the critical role of the home environment in setting healthy life trajectories from conception onwards.

What changed was not the environmental health concerns we were prioritizing for action, but how we framed the issues and who we engaged with to define both problems and solutions.

### The creation of RentSafe

A typical CPCHE health promotion project starts with emergent scientific knowledge about the health implications of a toxic substance(s), and a desire to equip frontline professionals with digestible information that they can incorporate into routine educational practices to support informed decision making. Put simply, it is about moving scientific knowledge into people’s everyday lives.

With RentSafe, EP and the CPCHE partners began in an entirely different way. We set our sights, not on edifying community members with precautionary environmental health advice, but on instigating an examination of upstream drivers of unhealthy housing conditions chronically experienced by marginalized populations. We began exploring the constellation of institutions, regulations, and professional norms that constitute the intersectoral “system” to find out why and how it is falling short. Given CPCHE’s predisposition to health equity concerns, this new orientation was not a source of controversy among the partners. It did, however, require a concerted refocusing and many conversations about the vision and methods of the nascent RentSafe project. RentSafe’s almost instant success in eliciting enthusiasm and buy-in from others helped to solidify CPCHE’s commitment to this new way of working.

In embarking on RentSafe, CPCHE and an evolving team of collaborators were beginning to engage in a form of collective reflexivity that is not without risks. We began shining a diagnostic spotlight into the professionalized realm in which many of us received our training and in many cases our employment or funding to find out where there are blind spots to be exposed, understood, and ultimately challenged. We were moving off the well-worn path of conventional knowledge translation into highly diversified intersectoral spaces where social justice questions loom large, and where the scientific terminology and evidence-based metrics to which we are accustomed were no longer the tools we most needed.

In keeping with an equity focus, CPCHE and the RentSafe team needed to bring together a full array of perspectives on housing habitability, including the views of people whose grounded expertise could offer a critical gaze into the functioning and failings of the intersectoral “system.” As such, our first step was to convene focus groups with 80 tenants living on low income in both urban and rural regions of Ontario.^1^ This achieved two things. First, focus group participants shed important light on the consequences of living in unfit housing and the experience of trying to get help. Second, the focus group events, purposefully organized with community partners, enabled the start of relationship-building with tenant advocates. The stories, perspectives, and ideas for change shared by tenants in the focus groups and the ongoing interaction with those who chose to join the RentSafe initiative provided the basis of our collective critique of the intersectoral system.

From there, the RentSafe team began to engage with relevant professional sectors—public health units, legal aid clinics, frontline workers in health and social services, municipal inspectors, and small-scale landlords—on housing habitability concerns and their capacity to respond. Together with sector-specific partners, the RentSafe team conducted a series of surveys of professionals in these sectors to better understand their capacities and challenges in addressing unfit rental housing conditions, including the effectiveness of referrals and other intersectoral interactions. The team then convened an intersectoral RentSafe Roundtable to work through the implications of what we were learning, including the gaps and disconnects that were coming to light (Phipps et al. 2016).

Positioning tenants with lived experience in leadership roles within RentSafe has significantly shaped how RentSafe partners have approached questions of housing habitability. A pivotal moment occurred when the tenant advocates, in their opening presentation at the RentSafe Roundtable, shifted the conversation from the dispassionate term “housing” to the human desire and right to have a home. Their ongoing leadership has ensured a holistic framing in which the pursuit of social justice and human dignity is inherent to RentSafe’s notion of habitability, and the meaning of “health” is at its broadest interpretation.

With RentSafe as its watershed moment, CPCHE is shifting from a top-down “for whom” orientation, in which scientific knowledge is translated for community-level uptake, to an authentically engaged “with whom” approach that enables intersectoral actors to reimagine the ways housing problems are defined and solutions constructed.

## Implications

Public health has long prioritized health equity as a core goal (WHO 1986, 2009). Important values reflected in Canadian public health core competencies include a commitment to equity, social justice and respect for diversity, self-determination, empowerment and community participation (PHAC 2008b). Despite this strong framework, public health practitioners remain challenged in their ability to reconcile these normative aspects of their work with the dominance of evidence-based thinking, often conflated with political neutrality, that itself has come under critique in recent years (Potvin and Jones 2011; Echt 2017; Gray and McDonald 2006; Masuda et al. 2008). This disconnect results in unrealistic attempts to address questions of health inequity equipped only with scientific evidence, to the exclusion of the social and cultural knowledge that can shed light on the unjust societal arrangements that produce such inequities in the first place.

Given the complexities inherent in health equity challenges, there is little debate that intersectoral approaches are needed, despite their challenges (Bilodeau et al. 2018). But how can we know whether our intersectoral processes are moving us towards a more justice-based public health?

What we as co-authors are calling equity-focused intersectoral practice (EquIP) may offer an approach to intersectoral work that integrates the critical thinking, drawn from the social sciences, that we believe is essential to supporting this shift in public health focus. Expanding on the EqKT framework, EquIP aims to counteract the tendency within public health to take a deficit view that defines populations based on their measured vulnerability, instead of calling for a reversal of the gaze into the institutional structures that perpetuate such vulnerabilities. This “reversing the gaze” requires public health and other professionals to undertake reflexive and relational effort that can be prompted by three key questions: What do I need to do to prepare? With whom am I working? and How am I working?

### What work do I need to do on myself before I enter spaces of intersectoral action?

This question asks public health professionals to engage in honest reflection on our own positionality, agency, and even complicity, in the system that is failing to resolve persistent health inequities. This is humble work that requires us to shed the protective mantle of professional expertise and subordinate our position to those who experience the consequences of environmental health inequities. This iterative (un)learning process cannot be achieved by reviewing statistics or listening to “stories of pain” (Tuck and Yang 2014). It is relational work that requires us, as actors on the inside of the intersectoral system, to put ourselves in situations and relationships in which our relative power comes into sharp relief. If you are feeling out of your element, you are on the right track.

In the creation of RentSafe, EP, JM, and other RentSafe team members were asking our professional partners to enter into an intersectoral space in which the voices of those whose lives bear the consequences of the societal failure to ensure housing habitability were at the centre of the room. We were asking tenants to courageously share their personal experiences and ideas for change with people from the institutionalized sectors that have, to varying degrees, failed them. We engaged in significant preparation, including one-on-one conversations as well as ground-setting events (e.g., a retreat-style meeting among our broadly diverse RentSafe Advisory Committee) to help orient people to the equity-focused and relational nature of the RentSafe initiative. We worked hard to create non-hierarchical and inclusive intersectoral spaces in which community members and professionals understood their dual roles as both teachers and learners. We reiterated often that people and relationships matter more than outputs.

### With whom do I seek to work?

Equity-focused intersectoral practice requires that professional actors pause to consider who we include in our definitions of relevant sectors, and whether there are gaps that we need to acknowledge and address. If our list includes only professionalized institutions and organizations, or if our marker of success is the number of organizations that have participated rather than the strength of relationships developed, it is time to rethink. EquIP should move us beyond thinking of who we have “reached out to” or “invited in,” to a question of whose trust we have earned to legitimately serve as collaborators and allies.

In the example of RentSafe, applying an equity-focused lens has expanded CPCHE’s collaborative relationships beyond the formal set of CPCHE partner and affiliate organizations to include tenants, housing providers, community food centres, Indigenous leaders, and community organizers, among others. While these more diversified engagements remain specific to the RentSafe project, CPCHE now has a strong precedent for rethinking with whom we need to engage in the initiation and implementation of future work. Our experiences in RentSafe have helped us to realize our shortcomings, including the important perspectives that are not yet represented at the core CPCHE table.

### How do we work together?

Various oppressions—class-based, racialized, gendered, or colonial—are tangled up within, and perpetuated by, institutional rules and professional practices. To work against these oppressions requires us to pay careful attention to the intersectoral spaces we seek to create. For example, some venues (e.g., government offices, conference venues) may be unwelcoming for some participants. Assumptions about literacy and familiarity with technology may inadvertently exclude some people from full participation. The pace of work of professional collaborations may come across as insensitive, while also not acknowledging the value of time invested by those not representing a professional role. And while public health carries an important role in convening intersectoral processes, there are transformational benefits when leadership is ceded to those whose grounded expertise offers vital knowledge with which to confront the issues at hand.

These considerations were reflected in a number of decisions made within RentSafe, including the use of arts-based and social justice venues instead of conference rooms, and the casual dress code established for all events. The RentSafe team took seriously the importance of acknowledging the land on which we were convening, and benefited from the wisdom of Elders and Indigenous knowledge keepers, who were integral to the agenda from start to finish, whenever possible. We placed a high priority on unstructured time during meetings to allow relationships to develop and new ways of thinking to emerge from the diversity of viewpoints and expertise around the table.

### Time to define competencies for equity-focused intersectoral practice?

A key challenge for public health is to embrace the normative nature of equity-focused work, which in turn requires us to confront the perception that critical examinations distract from, rather than support, the attainment of population health goals (Potvin and Jones 2011). If indeed the roots of health are societal, it is imperative to seek the transformation of societal conditions through robust intersectoral and participatory engagement, and to work in allyship as advocates (Hancock 2015). Drawing upon the experiences described in this paper, we contend that the ability of public health practitioners to fulfill this role requires us to develop reflexive skills, including inclusivity, humility, and transparency, as a necessary bridge between public health values and its core competencies. Our experiences suggest that working with a reflexive lens helps us to see beyond the so-called deficits of marginalized communities to gain a clearer view of the structural and practice-based changes needed to counteract enduring colonialism, patriarchy and class-based prejudice. Perhaps more importantly, the very process of engaging in equity-focused intersectoral practice can help us to transform ourselves, thereby enriching the human experience and helping us shift our path towards a justice-based pursuit of health equity goals.

## Footnotes

1 The current scope of RentSafe, funded by the Ontario Trillium Foundation, is Ontario.
